# Risk Factors for Addiction and Their Association with Model-Based Behavioral Control

**DOI:** 10.3389/fnbeh.2016.00026

**Published:** 2016-03-17

**Authors:** Andrea M. F. Reiter, Lorenz Deserno, Tilmann Wilbertz, Hans-Jochen Heinze, Florian Schlagenhauf

**Affiliations:** ^1^Max Planck Fellow Group ‘Cognitive and Affective Control of Behavioral Adaptation,’ Max Planck Institute for Human Cognitive and Brain SciencesLeipzig, Germany; ^2^Lifespan Developmental Neuroscience, Department of Psychology, Technical University of DresdenDresden, Germany; ^3^Department of Psychiatry and Psychotherapy, Campus Charité MitteCharité - Universitätsmedizin Berlin, Germany; ^4^Department of Neurology, Otto-von-Guericke UniversityMagdeburg, Germany; ^5^Department of Behavioral Neurology, Leibniz Institute for Neurobiology, Otto-von-Guericke UniversityMagdeburg, Germany

**Keywords:** decision-making, instrumental control, addiction, alcohol, family history, risk, impulsivity, cognitive capacity

## Abstract

Addiction shows familial aggregation and previous endophenotype research suggests that healthy relatives of addicted individuals share altered behavioral and cognitive characteristics with individuals suffering from addiction. In this study we asked whether impairments in behavioral control proposed for addiction, namely a shift from goal-directed, model-based toward habitual, model-free control, extends toward an unaffected sample (*n* = 20) of adult children of alcohol-dependent fathers as compared to a sample without any personal or family history of alcohol addiction (*n* = 17). Using a sequential decision-making task designed to investigate model-free and model-based control combined with a computational modeling analysis, we did not find any evidence for altered behavioral control in individuals with a positive family history of alcohol addiction. Independent of family history of alcohol dependence, we however observed that the interaction of two different risk factors of addiction, namely impulsivity and cognitive capacities, predicts the balance of model-free and model-based behavioral control. *Post-hoc* tests showed a positive association of model-based behavior with cognitive capacity in the lower, but not in the higher impulsive group of the original sample. In an independent sample of particularly high- vs. low-impulsive individuals, we confirmed the interaction effect of cognitive capacities and high vs. low impulsivity on model-based control. In the confirmation sample, a positive association of omega with cognitive capacity was observed in highly impulsive individuals, but not in low impulsive individuals. Due to the moderate sample size of the study, further investigation of the association of risk factors for addiction with model-based behavior in larger sample sizes is warranted.

## Introduction

Drug addiction tends to run in families and relatives of drug-dependent individuals have an eight-fold increased risk of developing addictive disorders compared with the general population (Merikangas et al., 1998). Endophenotype accounts of addiction postulate that unaffected relatives share alterations in behavioral or cognitive processes similar or intermediate to those observed in addicted individuals (Robbins et al., 2012).

Inspired by a rich body of work in cognitive neuroscience, recent developments in addiction research highlight a shift from goal-directed toward habitual instrumental control systems as biasing addicted individuals to repeatedly choose certain maladaptive behaviors even in the face of negative consequences (Redish, 2004; Everitt and Robbins, 2005; Redish et al., 2008; Dayan, 2009; Hogarth et al., 2013). Indeed, there is recent evidence from human patient studies pointing toward reduced goal-directed control in addiction (Hogarth, 2011; Hogarth and Chase, 2011; Sjoerds et al., 2013; Sebold et al., 2014; Voon et al., 2015).

This view on addiction as a shift from goal-directed toward habitual instrumental control builds upon the prominent notion that instrumental control in healthy decision-making arises from contributions of both a deliberative, goal-directed, and a reflexive, habitual system (Balleine and Dickinson, 1998; Dolan and Dayan, 2013). Learning algorithms have amended this theory by assessing possible underlying computations (Daw et al., 2005): on the one hand, goal-directed behavior, as formalized in “model-based” algorithms, uses a mental model of the environment; future actions and potential outcomes are planned in a forward manner and these costly computations enable flexible behavioral adaptation. On the other hand, habitual behavior, as described in “model-free” algorithms, is retrospective and rigid, but computationally efficient. It relies on “stamped-in” past rewards and neglects environmental structure.

A shift from goal-directed or model-based toward habitual or model-free behavior has not only been suggested for addiction itself, but also for recognized risk factors for addiction like acute and chronic stress (Schwabe and Wolf, 2009, 2010, 2011a,b; Schwabe et al., 2011a,b; Otto et al., 2013a; Radenbach et al., 2015) or impulsivity (Hogarth, 2011; Hogarth et al., 2012a; Deserno et al., 2015a). Studies in healthy at-risk populations are of particular importance, as they help to elucidate whether a shift toward model-free instrumental control precedes the development of addiction or is a consequence of addictive behavior. Further, they help to rule out potential confounders like neurotoxic effects on brain structure and globally impaired cognitive functioning. In particular, interindividual differences in cognitive functioning were shown to be associated with the degree of model-based control in healthy individuals (Otto et al., 2013a,b; Schad et al., 2014) but also with impairments in patients (Sebold et al., 2014).

In this study, we asked whether healthy individuals with a positive family history of alcohol-dependence show a bias toward model-free control as has been observed in addicted individuals. Building on previous evidence pointing toward an important role of impulsivity and cognitive capacity in instrumental control within populations at risk for or suffering from addiction (Ersche et al., 2012; Sebold et al., 2014), the study was also designed to assess these factors as additional moderators of behavioral control.

## Materials and methods

### Participants

20 healthy participants with a positive family history of alcohol dependence were recruited based on the CAST-6 (Children of Alcoholics Screening Test; Hodgins et al., 1993). We used Limesurvey (https://www.limesurvey.org/) to send a digital version of this self-report questionnaire to all members of the participant database of the Max Planck Institute for Human Cognitive and Brain Sciences for whom an e-mail address was available. *N* = 1260 participants answered the CAST-6 questionnaire. Only individuals with a score ≥ 5 were included [usually a score of score of ≥ 2 indicates a positive family history (Hodgins et al., 1993)] in the positive family history sample. In the lab, participants were additionally interviewed on parental alcohol consumption, confirming their fathers' fulfillment of DSM-IV criteria of addiction. To exclude any influence of potential prenatal alcohol exposure, only individuals with a father suffering from alcohol dependence were included. Seventeen healthy participants without a positive family history of alcohol use disorders (CAST-6 score of zero and no indication of any substance abuse for 1st–3rd degree relatives in a personal interview) were included as a control group. Both groups did not differ in age or gender distribution and were screened for axis-1 psychiatric disorders using the SCID-IV interview (First et al., 1997) and for presence of alcohol-related disorders in family members (up to 3rd degree). We had originally invited 22 participants for each experimental group. Upon arrival in the laboratory, all participants underwent the above-described diagnostic procedure. Based on that, seven participants were excluded due to fulfillment of the following criteria: history or presence of severe psychiatric symptoms or regular illegal drug consumption as indicated by the SCID interview (*n* = 3), pregnancy in the last trimester (*n* = 1), color blindness (*n* = 1), and diagnosis of alcohol dependence in family members other than their father (*n* = 2 in the control group). Thus, none of the participants included in our analyses fulfilled criteria of an axis-1 disorder at the time of the study. None of the included control participants reported alcohol-related disorders in family members (1st–3rd degree).

To further characterize the sample, all participants underwent neuropsychological assessment in the form of four tests: the Digit Symbol Substitution Test (DSST; Wechsler, 1955) and the Reitan Trailmaking Test (TMT; Reitan, 1955) part A for processing speed, the TMT part B for complex attention/executive function and the Backward Digit Span Test (Wechsler, 1955) for working memory. All test scores were z-transformed and z-transformed scores of all four tests were averaged for a composite measurement of cognitive capacities (compare Schlagenhauf et al., 2013). Crystallized intelligence was examined based on a German vocabulary test (Schmidt and Metzler, 1992). In addition, participants completed the BIS-11 (Patton et al., 1995; Stanford et al., 2009), a well-established measurement to assess trait impulsivity. Participants also indicated alcohol consumption in the preceding 4 weeks using the Time Line Follow Back questionnaire (Sobell and Sobell, 1992). For a detailed group description, please see Table 1. The study was approved by the ethics committee at the medical faculty of the University of Leipzig and written informed consent was obtained from all volunteers. Participants were reimbursed on an hourly basis.

**Table 1:** **Sample characteristics of the original sample**.

	**With positive family history of alcohol dependence (*N* = 20)**	**With negative family history of alcohol dependence (*N* = 17)**	**Sig**.
Age (years)	28.65 ± 5.76 (19–42)	29.24 ± 5.47 (21–41)	0.76
Gender	10 female/10 male	8 female/9 male	0.86
DSST (19/16)	83.89 ± 10.55 (70–105)	86.75 ± 16.76 (57–120)	0.54
TMT A (19/16)	26.62 ± 8.55 (12–45)	20.46 ± 6.13 (9–31)	0.02
TMT B (19/16)	54.33 ± 26.92 (30–95)	51.06 ± 19.73 (16–88)	0.61
Digit span (19/16)	7.95 ± 2.48 (4–13)	8.06 ± 2.77 (4–14)	0.90
Z-score fluid IQ (19/16)	0.14 ± 0.66 (−1.33–0.91)	0.16 ± 0.89 (−1.61–1.83)	0.28
Verbal Intelligence (19/16)	109.79 ± 9.31 (92–129)	112.38 ± 9.14 (97–133)	0.42
BIS total (18/16)	60 ± 7.76 (49–73)	59.63 ± 7.51 (45–74)	0.89
CAST-6	5.70 ± 0.47 (5–6)	0	–
Time-Line-Follow-Back (18/17)	19.39 ± 17.97 (1–70)	20.32 ± 24.81 (0–98)	0.90

*Group means with standard deviations and range in brackets are reported; for group comparisons two-tailed two-sample t-test or Chi-Square Tests were used*.

### Sequential decision-making task

**Figure 1:**
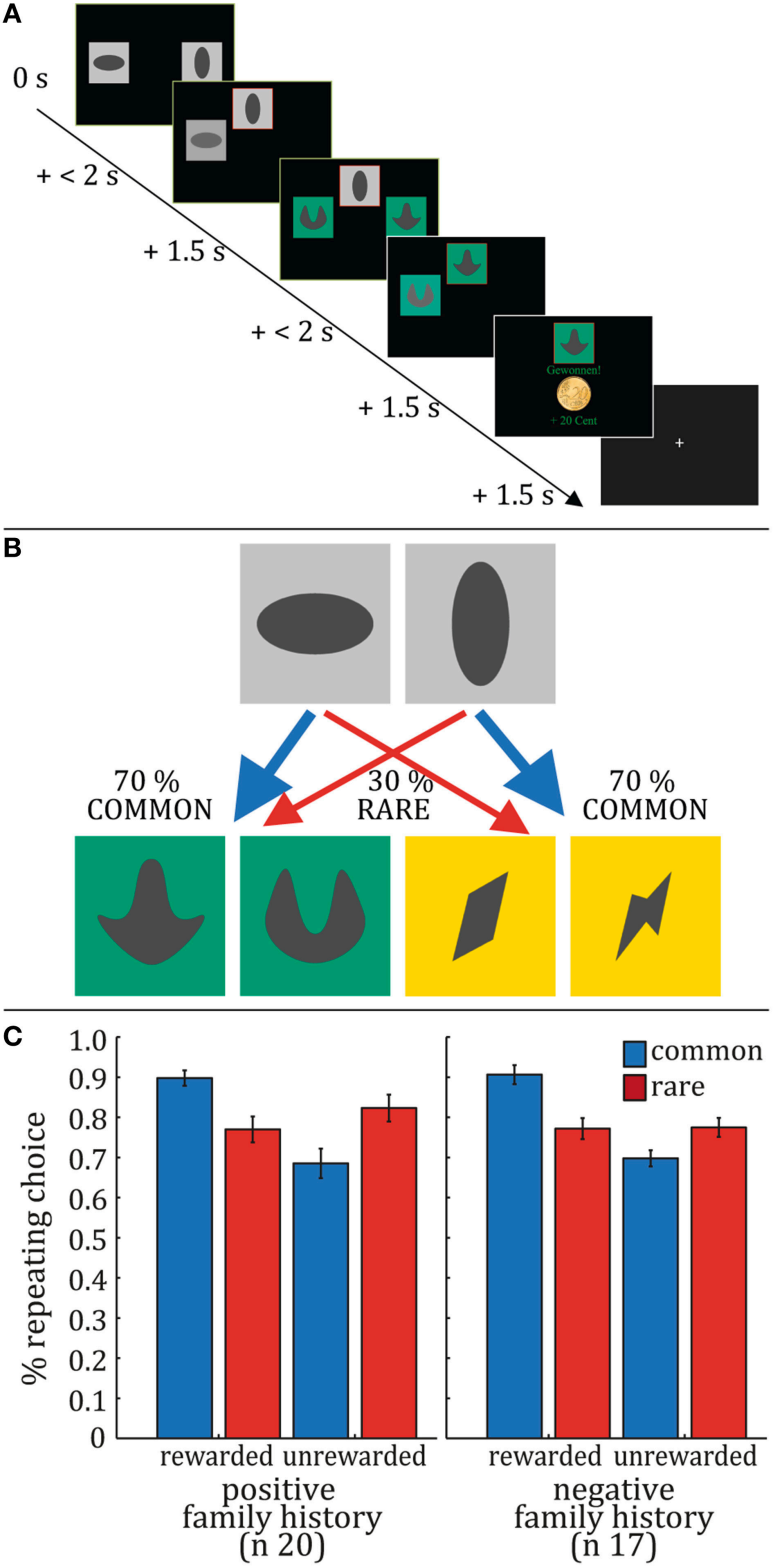
**Task and Raw Data Results**. **(A)** Exemplary trial sequence. At each stage, subjects made a choice (maximum decision time 2 s) between two stimuli presented: two gray boxes at the first stage and two pairs of differently colored boxes at the second stage. After this choice the respective stimulus was framed in red, moved to the top of the screen and remained there for 1.5 s. before the subject entered the second stage, where another choice had to be made. Reward was delivered after the second-stage choice. **(B)** First and second stage choices were linked via a fixed transition probability: each first-stage choice led to one pair of the second-stage stimuli with a probability of 70% **(C)**. Stay-switch behavior at the first-stage of the task was analyzed as a function of reward and state in the previous trial. These stay probabilities were subjected to repeated-measures ANOVAs with reward and state as within-subject factors and group as a between-subject factor. We observed a significant main effect of reward (*F* = 23.66, *p* < 0.001) and reward × state interaction (*F* = 43.83, *p* < 0.001); no significant main effect of state (*F* = 0.95, *p* = 0.34) and no significant reward × group (*F* = 0.38, *p* = 0.54), state × group (*F* = 1.85, *p* = 0.18) or reward × state × group (*F* = 0.57, *p* = 0.46) interactions could be observed.

A two-step choice task was implemented as in previous studies (e.g., Daw et al., 2011; Deserno et al., 2015a,b). The task consisted of 201 trials; each trial involved two choice stages. At each stage, subjects were required to give a forced choice (maximum decision time 2 s) between two stimuli presented; stimuli were two gray boxes at the first stage and two pairs of differently colored boxes at the second stage (Figure 1). The position on the screen where stimuli were presented (left vs. right) was randomized over trials. After a choice the respective stimulus was framed in red and moved to the top of the screen, where it remained for 1.5 s. Rewards were delivered only after the second-stage choice. The reward probabilities of second-stage stimuli were identical to Daw et al. (2011). First and second stage choices were connected via a fixed transition probability: each first-stage choice was associated with one pair of the second-stage stimuli via a fixed probability of 70% (Figure 1). Each trial was ended by an exponentially distributed inter-trial interval (ITI) with a mean of 2 s.

During an instruction session prior to the experiment, participants were explicitly informed that the transition structure would not change throughout the task. Participants were also told about the independence of reward probabilities and their dynamic change over the course of the experiment. Participants were instructed to win as much money as possible and informed that the balance of their account would be paid out in addition to the reimbursement for study participation. After detailed instruction including teach-back, participants trained on a shortened version of the task (50 trials) with different reward probabilities and stimuli.

Note that inference on model-based vs. model-free control strategies in this task is made based on the choices observed at the first stage of the task. The rationale is that a learner can be influenced by two sources of information to come to a decision at the first stage. Firstly, decisions at the first stage can be influenced by rewards obtained after the second stage. Relying on these previously experienced rewards, in the sense of repeating choices in the future which had led to a reward in the past, would correspond to a habitual or model-free strategy. As a second source of information, the learner can take the previously acquired task structure into account. In the case of this task, the task structure corresponds to the transition probabilities between the two stages. Practically, this would mean repeating decisions at the first stage based on an if-else pattern: the first-stage choice should only be repeated if the second-stage choice was rewarded *and* occurred under the common (i.e., 70% probability) transition state. In the case where the second-stage choice was rewarded but the coupling of the first and second stage stimulus pair was untypical and rare (i.e., 30% probability), the first stage choice ought—despite a (rather accidently) obtained reward for the choice sequence—not be repeated. Combining learning from rewards with the knowledge of the transition matrix would correspond to a model-based strategy, which takes into account the environmental transition structure when making decisions. Thus, analysis of first-stage choices as a function of reward and state (rare vs. common transition) allows for inferences about the contribution of model-based and model-free strategies to participants' decision behavior.

### Behavioral data analysis

Data were analyzed using MATLAB R2012 and Statistics Toolbox Release 2012b (The MathWorks, Inc., Natick, Massachusetts, United States), IBM SPSS Statistics for Windows, Version 22 (IBM Corp., Armonk, NY) and R (R Foundation for Statistical Computing, Vienna, Austria, http://www.R-project.org/).

Stay-switch behavior on the first step was analyzed as a function of reward (reward or no reward) and state (common or rare) of the previous trial. Individual stay probabilities were subjected to a repeated-measures ANOVA with reward and state as within-subject factors and group as a between-subject factor.

### Computational modeling

The aim of model-free and model-based algorithms is to learn values for each of the stimuli, which appear in the task as three pairs (s_A_, s_B_, s_C_). s_A_ refers to the first-stage stimuli. Importantly, for the first stage, values derived from the model-free and model-based algorithms differ. s_B_ and s_C_ refer to the two pairs of second-stage stimuli. *a* refers to the chosen stimulus. The index i denotes the two stages of the task (*i* = 1 for *S*_*A*_ at the first stage and *i* = 2 for *S*_*B*_ *or S*_*C*_ at the second stage) and the index *t* denotes the trial.

First, the model-free algorithm was SARSA(λ) (state-action-reward-state-action; Sutton and Barto, 1998). Based on temporal-difference (TD) prediction errors, this algorithm makes predictions about state-action pairs and thus tracks the value for each combination of state (here, the stimuli pairs: s_A_, s_B_, and s_C_) and choice of the participant (action *a*) over the course of learning.

(1) $$\begin{array}{l} Q_{MF}\left(s_{i,t+1},a_{i,t+1}\right)=Q_{MF}\left(s_{i,t},a_{i,t}\right)+\alpha_{i}\delta_{i,t} \end{array}$$

These values are updated on a trial-by-trial basis via a teaching signal, the reward prediction error δ. The reward prediction error captures the difference between the anticipated and actually received reward:

(2) $$\begin{array}{l} \delta_{i,t}=r_{i,t}+Q_{MF}\left(s_{i+1,t},a_{i+1,t}\right)-Q_{MF}\left(s_{i,t},a_{i,t}\right) \end{array}$$

Notably, *r*_1, *t*_ = 0, because no reward is delivered after a first-stage choice. Further, we introduce an additional parameter λ. λ accounts for the possibility that learners' decisions on the first-stage are influenced by reward prediction errors experienced at the second stage. It thus connects the two stages of the task:

(3) $$\begin{array}{l} Q_{MF}\left(s_{1,t},a_{1,t}\right)=Q_{MF}\left(s_{1,t},a_{1,t}\right)+\alpha_{1}\lambda \delta_{2,t} \end{array}$$

Note that λ additionally reflects the main effect of rewards (delivered after the second stage's decision) on decisions at the first stage as observed in the raw data analysis of stay-switch behavior. Crucially, it does not reflect an interaction of reward and state (and thus, usage of the task structure) on raw data choice behavior. Instead, the interaction of reward × state would require representation of values for the transition matrix (in the sense of learning an if-else-pattern when mapping states to actions). TD learning represents a principled and theory-grounded basis of habitual learning as it requires outcome experience to retrospectively update choice values, which substantially slows adaptation. Nevertheless, it is noteworthy that it does not map directly on a habit, which represents automatic behavioral responses to a stimulus divorced from its recent outcome value.

The model-based algorithm takes the transition matrix—and thus the environmental structure of the task—into account. It does so via computing first-stage values by multiplying maximum values (*max Q*) at the second stage [derived from model-free learning as described in formula (3)] with transition probabilities:

(4) $$\begin{array}{l} Q_{MB}\left(s_{A},a_{j}\right)=Q_{MB}\left(S_{B}|S_{A},a_{j}\right)\;max\;Q_{MF}\left(s_{B},a\right)\\ \;+Q_{MB}\left(S_{C}|S_{A},a_{j}\right)\;max\;Q_{MF}\left(s_{c},a\right) \end{array}$$

Note that in this approach the transition probabilities are not learned explicitly. Rather, it tests whether participants *use* the task structure (which they have acquired beforehand). This approach is in line with the task instructions where participants enter the experimental task only after having trained on the transition probabilities in an intense training session. Daw and colleagues report a simulation which verified that this approach outperforms incremental learning of the transition matrix (Daw et al., 2011).

Third, the hybrid algorithm connects model-free and model-based learning via the weighting factor ω:

(5) $$\begin{array}{l} {Q\left(s_{A},a_{j}\right)=\omega Q}_{MB}\left(s_{A},a_{j}\right)+\left(1-\omega \right)Q_{MF}\left(s_{A},a_{j}\right) \end{array}$$

Importantly, ω reflects the relative influence of model-free and model-based values on participants' choices. According to formula (5), lower values of ω (0 < ω < 0.5) indicate that the learner relies more on the reward-driven, model-free strategy to solve the task. If ω = 0, the learner fully relies on model-free computations and neglects the transition matrix. On the contrary, higher values of ω (1 < ω > 0.5) assign more weight to model-based computations, taking into account the transition probabilities as the central environmental feature of the task. In the case of ω = 1, the learner fully relies on model-based computations.

Consequently, a value of 0.5 indicates balanced contributions of both the model-free and the model-based system to choice behavior. For the analysis at hand ω is therefore the parameter of most interest, representing the balance and possibly also interindividual differences in the recruitment of the two decision-making systems.

The values derived from the algorithms described above are transformed into action probabilities using a softmax function for the value Q:

(6) $$\rho \left(a_{i,t}=a|s_{i,t}\right)=\frac{exp(\beta_{i}[Q(s_{i,t},a)+\rho *rep(a)])}{\sum_{a^{\prime }}exp(\beta_{i}[Q(s_{i,t},a^{\prime })+\rho *rep(a^{\prime })])}$$

This choice rule includes additional parameters β_i_, separately for both stages i. β controls the stochasticity of the choices. Put differently, with higher values of β, it is more likely that the learner takes the action with the highest expected value, thus, choice behavior is more tightly determined by the learning model. On the other hand, the lower the value of β, the less likely it becomes that the learner chooses the action suggested by the model. Thus choices are less influenced by the model or more stochastic. Separate β for both stages are estimated as the degree of stochasticity is assumed to be different between the two stages. The additional parameter ρ captures first-stage choice perseveration and *rep* is an indicator function that equals 1 if the previous first-stage choice was the same. This parameter accounts for strong perseveration at the first stage as observed in this task. In summary, the algorithm totals seven parameters. It can be reduced to its special cases ω = 1 (four parameters) and ω = 0 (five parameters).

### Model fitting

To infer the maximum-a-posteriori estimate *MAP* of parameters θ, we use a Gaussian prior with mean and variance μ and σ:

(7) $$\begin{array}{l} {MAP}_{i}=\text{argmax}\log p\left(Y|\theta \right)\;p\left(\theta |\mu ,\sigma \right) \end{array}$$

where Y represents the data in terms of actions A_*i*_ per subject *i*. We set priors empirically to the maximum likelihood estimates *ML* of μ and σ given the data by all subjects:

(8) $$\begin{array}{l} {ML}_{i}=\text{argmax}\log p\left(Y|\theta \right) \end{array}$$

and achieve this by using Expectation-Maximization. For an in-depth description please compare Huys et al. (2011, 2012).

All seven parameters of the best-fitting model were subjected to a multivariate ANOVA with group (family history: positive/negative) as a between-subject factor. Constrained parameters were transformed to a logistic (α, λ, ω) or exponential (β) distribution to enforce constraints and to render normally distributed parameter estimates.

### Model selection

To compare models for their relative goodness of fit, we computed the model evidence by integrating out free parameters. This integral was approximated by sampling from the empirical prior distribution (Huys et al., 2011, 2012). The integrated likelihood was subjected to the spm_BMS function contained in SPM8 (http://www.fil.ion.ucl.ac.uk/spm/) to compute expected posterior probabilities and their exceedance probabilities (XP) (Stephan et al., 2009).

### Regression models and moderator analyses

To test the potential influence of impulsivity and cognitive capacities on the balance of model-based and model-free behavioral control, we built a linear regression model with ω as dependent variable and family history (positive vs. negative), impulsivity (BIS-11 total score), sum score across cognitive capacities as predictor variables. In all models, we additionally included age as nuisance variable as it is known to impact model-based behavior (Eppinger et al., 2013). Further, the negative log-likelihood of the hybrid model was included as independent variable to control for unspecific effects of individual variability in model fit. To test potentially interacting effects of the risk factors on ω, we applied moderator analyses (Hayes and Matthes, 2009).

## Results

### Behavioral raw data

As in previous studies with the same task (e.g., Daw et al., 2011; Deserno et al., 2015b), analysis of stay-switch behavior at the first-stage as a function of reward and state in the previous trial revealed a main effect of reward (*F*_(1, 35)_ = 23.657, *p* < 0.001) and a reward × state interaction effect on first-stage decisions (*F*_(1, 35)_ = 43.826, *p* < 0.001, Figure 1C). In individuals with a positive family history of alcohol dependence neither evidence for a reduction of model-based choices (reward × state × family history interaction *F*_(1, 35)_ = 0.570, *p* = 0.461, Figure 1C) nor for a shift toward model-free control (reward × family history interaction *F*_(1, 35)_ = 0.379, *p* = 0.542, Figure 1C), nor a main effect of group on stay/switch behavior (*F*_(1, 35)_ = 0.029, *p* = 0.864) was observed.

### Computational modeling

We compared three computational models: a model-based algorithm (ω = 1), a model-free algorithm (ω = 0) and a hybrid model with ω as a free parameter. Confirming previous studies with the same task and modeling analysis, model selection across all participants revealed that the hybrid model explained the observed choice behavior best (XP model-based = 0.029, XP model-free = 0.006, XP hybrid model = 0.965). See Table 2 for the distribution of the best-fitting parameters of the hybrid model. With respect to family history of alcohol dependence, we tested for between group differences by subjecting all seven parameters of the hybrid model to a multivariate ANOVA (mANOVA) with family history (positive/negative) as between-subject factor. There was no significant effect of group (*F*_(7, 29)_ = 0.760, *p* = 0.280). Thus, the mANOVA did not indicate any evidence to reject the null hypothesis that parameters of the model do not differ between the two experimental groups.

**Table 2:** **Distribution of best-fitting parameters (hybrid model)**.

	**Min**	**1st Qu**.	**Median**	**Mean**	**3rd Qu**.	**Max**
β_1_	2.56	5.09	6.47	7.02	8.23	14.11
β_2_	1.38	2.64	3.15	3.63	4.19	7.96
α_1_	0.07	0.36	0.50	0.51	0.67	0.83
α_2_	0.04	0.41	0.53	0.50	0.66	0.91
λ	0.23	0.49	0.65	0.62	0.78	0.93
ω	0.34	0.55	0.66	0.63	0.73	0.83
ρ	0.02	0.09	0.15	0.16	0.21	0.32
−LL	−280.60	−217.70	−188.80	−183.90	−156.80	−88.43

*Min, minimum; 1st/3rd Qu, first and third quartile; Max, maximum*.

### Evidence in favor of the null hypothesis

Traditional frequentist analyses do not allow to infer evidence in favor of the null- vs. the alternative hypothesis. By contrast, Bayes factors quantify the support that the data provide for the null hypothesis vis-a-vis the alternative hypothesis. Thus, to examine the suggested null finding in a *post-hoc* manner, we used JASP (Love et al., 2015) to compute one-sided Bayesian independent *t*-tests between groups (Rouder et al., 2009; Wetzels et al., 2009). A Cauchy prior with a width of *r* = 1 for the effect size of the alternative hypothesis was used (Rouder et al., 2009; Wetzels et al., 2009; Wagenmakers et al., 2015). As explained above, the balance between model-based and model-free control was of most interest in the analysis at hand. Indicative of this balance is the parameter ω and comparing this parameter showed moderate evidence in favor of the null hypothesis as indicated by a Bayes Factor B_01_ ~6 (see Table 3). A similar effect was present for the interaction term of reward × state in the raw data reflecting the degree of model-based control. A Bayes Factor B_01_ of 6 indicates that the observed data are six times more likely under the null hypothesis that participants with and without positive family history of alcohol-dependence do not differ with regards to the balance of model-based vs. model-free control. In addition, comparing the parameters of the hybrid model between groups revealed evidence in favor of the null hypothesis in most of the cases (as indicated by a Bayes Factor B_01_ of > 3 (Kass and Raftery, 1995). See Table 3 for results.

**Table 3:** **Results of Bayesian *t*-tests probing the hypothesis that model parameters and the interaction term of reward × state as a raw data indicator for the degree of model-based behavior is lower in participants with alcohol-dependent fathers as compared to participants without positive family history**.

**Bayesian Independent Samples** ***T*****-Test**		
	**B**_01_	**error (%)**
Interaction score raw data	6.691	~1.596e^−4^
β_1_	1.447	~1.751e^−4^
β_2_	1.838	~1.651e^−4^
α_1_	11.172	~4.376e^−6^
α_2_	4.290	~1.448e^−4^
λ	5.395	~1.489e^−4^
ω	5.933	~1.528e^−4^
ρ	7.781	~1.727e^−4^

*In the table, we show evidence in favor of the null hypothesis. By convention, a Bayes factor > 3 indicates evidence in favor of the hypothesis (Kass and Raftery, 1995). The Bayes Factor is interpretable as an Odds Ratio: for example, it is more than six times more likely that the data occurred under the null hypothesis that interaction of reward and state does not differ between groups than under the alternative hypothesis of a difference between groups*.

### Repeating the analyses with an increased sample size

As stated above, the sample size for this study was at the lower end. As one of our reviewers expressed concerns about the resulting lack of power to detect a true effect, we increased sample size in both groups. This was achieved by adding datasets, which had been acquired for other studies on different research questions but using the same task (subjects were taken from the following studies: Sebold et al., 2014; Deserno et al., 2015a,b; Radenbach et al., 2015 plus three unpublished datasets). For subjects included in these studies, information on family history of alcohol-dependence was available or could be obtained in a follow-up interview. All participants had been screened for substance-related disorders, and were excluded in case of any indication. Eight subjects with an alcohol-dependent father could be identified (*n* = 4 from Deserno et al., 2015a, *n* = 1 from Radenbach et al., 2015, *n* = 3 unpublished) and were matched with *n* = 11 participants without any family history of alcohol addiction (*n* = 7 from Sebold et al., 2014, *n* = 4 from Deserno et al., 2015b) in order to yield equal samples sizes. This resulted in an increased sample size of *n* = 28 gender- and age-matched participants per group (*n* = 11 female participants per group; negative family history group: mean age = 29.500 years, *SD* = 6.173; positive family history group: mean age = 29.393 years, *SD* = 7.325). Repeating the above described analyses did not yield any divergent results regarding model-based and model-free behavior: the reward × state × family history interaction did not show a significant effect (*F*_(1, 54)_ = 0.321, *p* = 0.575). The interaction of reward x group (*F*_(1, 54)_ = 0.331, *p* = 0.570) and the main effect of group were also not significant (*F*_(1, 54)_ = 0.722, *p* = 0.401). Further, we did not find any evidence for a group difference on the parameter ω from the computational modeling analysis (*t*_(1, 54)_ = .358, *p* = 0.722). In accordance with these results, which point toward a null finding, Bayesian independent *t*-tests on the raw data interaction score and the parameter ω revealed Bayes Factors of B_01_ = 7.316 and B_01_ = 6.405 in favor of the null hypothesis that the two groups do not differ in terms of model-based behavior.

### Cognitive capacities and impulsivity as potential moderator variables

As we did not observe alterations in the balance of model-free and model-based control to be associated with positive family history *per se*, we additionally tested for the influence of impulsivity as a risk factor associated with positive family history (Ersche et al., 2013) and cognitive capacities as they have been reported to moderate group effects on model-based behavior (Sebold et al., 2014). In a linear regression model with ω as dependent variable and family history, impulsivity, sum score across cognitive capacities as predictor variables, as well as age and the negative log-likelihood as nuisance variables, the effect of cognitive capacity reached significance (beta = 0.437, *t* = 2.076, *p* = 0.048) but there was no effect of the risk factors family history (beta = 0.186, *t* = 1.065 *p* = 0.296) or impulsivity (beta = 0.180, *t* = 1.031, *p* = 0.312).

**Figure 2:**
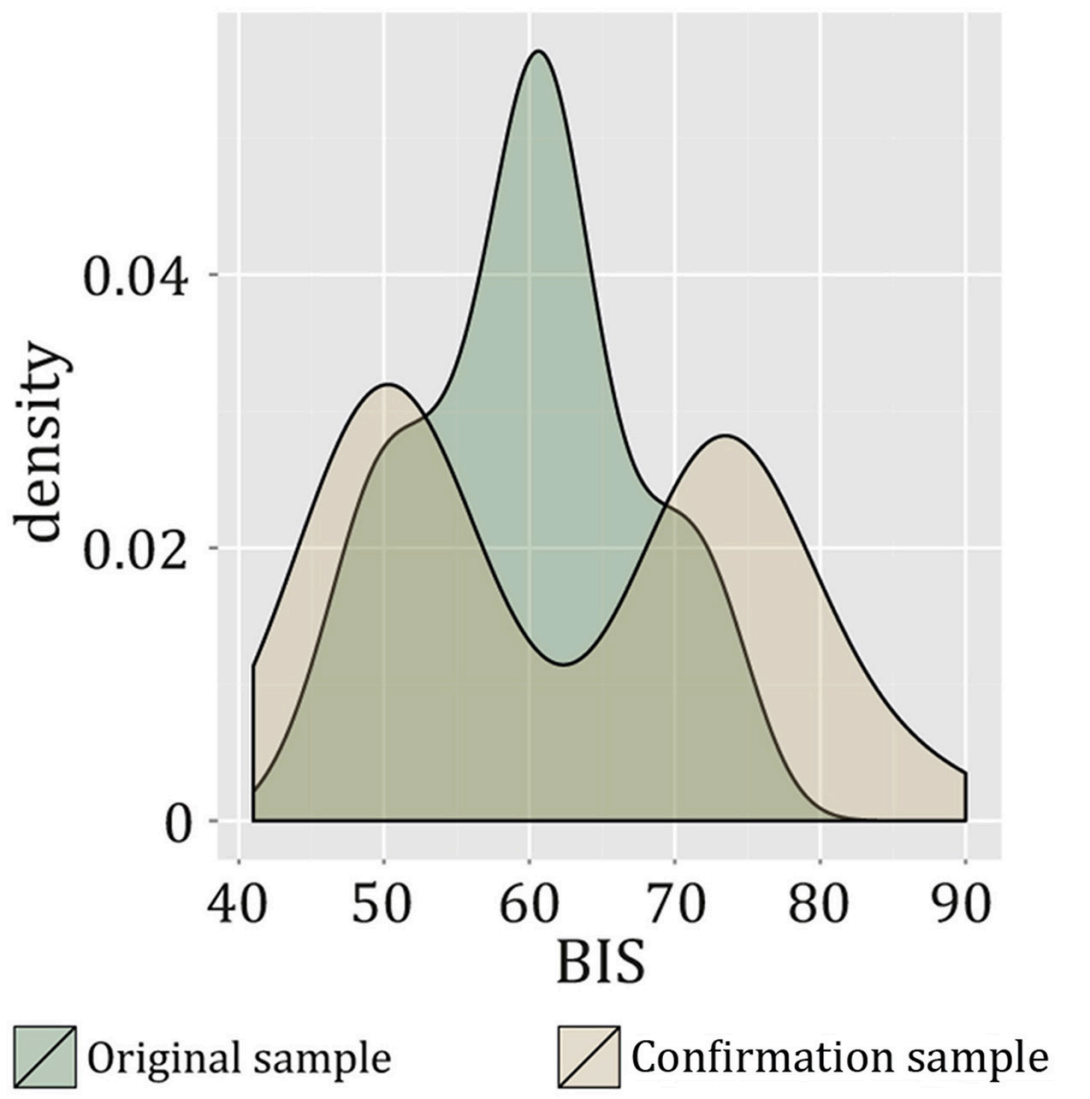
**Density function of BIS-11 values in the original sample and the confirmation sample**. The different distributions are due to differences in recruitment strategy: in the confirmation sample, participants were specifically chosen based on particularly low vs. high values on the BIS-11 (Deserno et al., 2015a).

**Figure 3:**
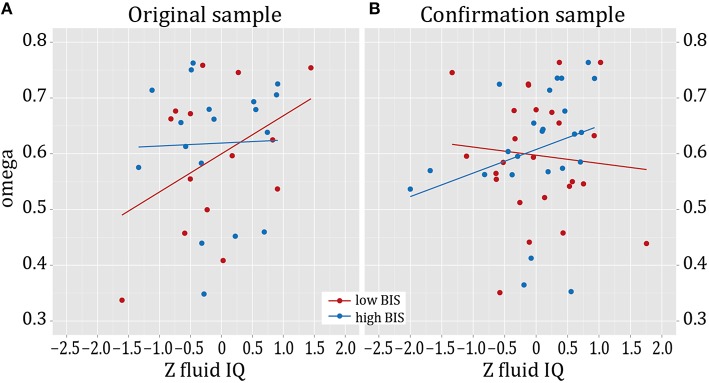
**Model-based behavior and cognitive capacity. (A)** Association of model-based behavior (as given by the parameter omega) with cognitive capacity (Z-score of fluid intelligence) in the lower, but not in the higher impulsive group of the original sample. **(B)** In the confirmation sample, a positive association of omega with cognitive capacity was found in the high-impulsive subgroup. In the original sample, high and low impulsive groups were defined based on a median split. In the confirmation sample, groups were defined by sampling from the upper and lower ends of the BIS-11 range in a larger sample (*n* = 452) according to their particularly high vs. low values in the BIS-11 (Deserno et al., 2015a).

Next, we aimed to probe whether the effect of cognitive capacities on model-based control is moderated by the two risk factors family history and impulsivity, respectively. See Figure 2 for the distribution of impulsivity scores in this sample and the subsequently described confirmation sample. In the respective moderator analyses, the interaction between positive family and cognitive capacities did not show a significant effect (R^2^-change due to interaction = 0.002, *F* = 0.058, *p* = 0.810), whereas the interaction effect between impulsivity (BIS score) and cognitive capacities on ω was significant (R^2^-change due to interaction = 0.127, adjusted R^2^-change due to interaction = 0.122, *F* = 5.256, *p* = 0.030, Figure 3A). To account for a potential effect of outliers on the observed findings, we performed a robust regression analysis with a bisquare reweighting function and confirmed the significant finding (beta = −0.338, *t* = −2.158, *p* = 0.040). Note that as group was included as a regressor in the model, the analysis accounts for possible influences of positive family history on this interaction effect of cognitive capacity and impulsivity on ω.

**Figure 4:**
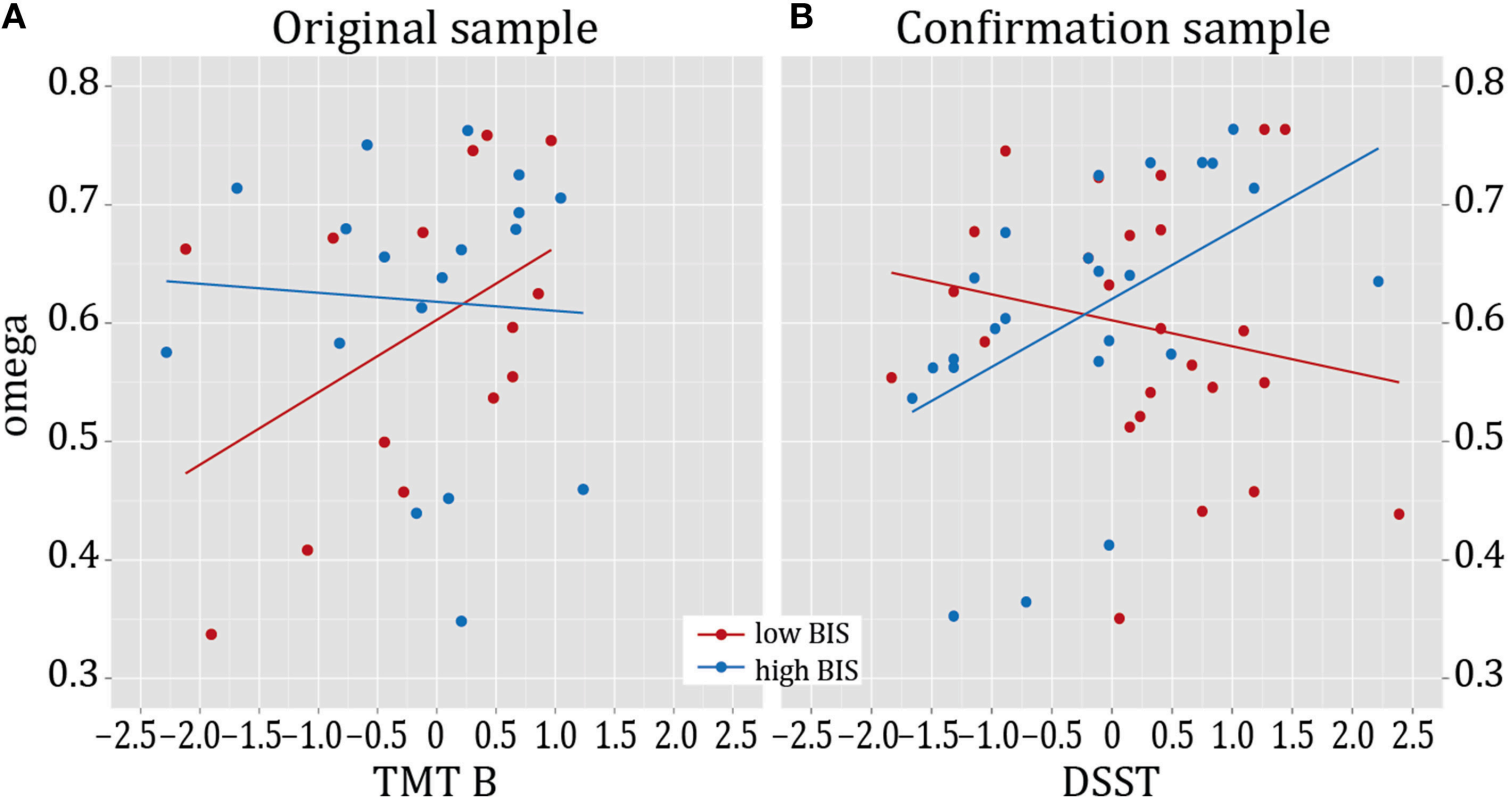
***Post-hoc* tests with cognitive subdomains. (A)** In the original sample, omega correlates positively with TMT B in the low impulsivity group. **(B)** In the confirmation sample, omega correlates positively with DSST scores in the high impulsivity group. In the original sample, high- and low-impulsive groups were defined based on a median split. In the confirmation sample, groups were defined by sampling from the upper and lower ends of the BIS-11 range in a larger sample (*n* = 452) according to their particularly high vs. low values in the BIS-11 (Deserno et al., 2015a). We plot z-transformed scores of the cognitive test scores.

We also explored the included neurocognitive subdomains by subsequently entering the four different test scores (TMT A, TMT B, DS, DSST) as independent variable in separate moderator analyses (dependent variable ω, moderator variable impulsivity). This revealed a positive effect of executive control (TMT B) (R^2^ change due to interaction = 0.136, adjusted R^2^ change due to interaction = 0.131, *F* = 5.348, *p* = 0.029) on the association of impulsivity and model-based behavior, whereas the other cognitive subdomains failed to contribute significantly to an interaction effect (TMT A: *F* = 2.588, *p* = 0.119, DS: *F* = 3.310 *p* = 0.0763, DSST: *F* = 2.900, *p* = 0.100, R^2^ changes due to interaction ≤ 0.090). We explored this interaction effect in a *post-hoc* fashion by using the median of the BIS score to split our sample in a subgroup with higher vs. lower trait impulsivity scores. The regression analysis was then repeated for those groups separately. We observed that the interaction effect between impulsivity and executive control was driven by a significant effect of executive control on ω in the lower impulsive subgroup (beta = 0.591, *t* = 2.574, *p* = 0.024). There was no association of executive control and ω in the higher impulsive subgroup (beta = −0.110, *t* = 0.341, *p* = 0.738). See Figure 4 for an illustration.

### Cognitive capacities, impulsivity, and model-based choices: Confirmation analysis in an independent sample

Using the same task and computational modeling analysis, we previously investigated the association between high vs. low trait impulsivity (defined according to BIS-11) and the balance between model-based and model-free decision-making (Deserno et al., 2015a). In this independent study, young (age range: 20–33 years) and healthy participants were recruited via the participant database of the Max Planck Institute for Human Cognitive and Brain Sciences: fifty healthy participants were drawn from the upper and lower ends of 452 individuals completing the Barratt Impulsiveness Scale online and invited to the lab. Using fMRI and the above-described sequential decision-making task together with the computational modeling analysis as applied here, the study was designed to compare high- vs. low-impulsive individuals regarding behavioral and neural signatures of model-based and model-free behavioral control. In short, no behavioral evidence for an influence of high vs. low trait impulsivity on the parameter ω was found. For a detailed description of recruitment strategy, sample characteristics and results, please compare Deserno et al. (2015a). Given the above reported findings, we now reanalyzed these data with respect to an interaction effect of impulsivity and cognitive capacity on ω, an analysis that had not been performed in the original investigation. All datasets included in the previous investigation (*n* = 50, 24 high-impulsive and 26 low-impulsive participants) were reanalyzed for confirmation. It is important to note that in the original study, participants were selected from the upper and lower ends of the BIS-11 range in a larger sample (*n* = 452) according to their particularly high vs. low values in the BIS-11. According to the previous literature (Stanford et al., 2009), mean total BIS-scores of each group met the criteria for high or low impulsiveness, respectively. This difference in the study design results in a different distribution of total BIS scores in the confirmation sample from Deserno et al. (2015a) as compared to the original sample of participants with and without positive family history of alcohol-dependence (compare Figure 2). Specifically, the respective high- vs. low-impulsive subgroups of both samples were significantly different from each other (comparing lower impulsive groups of the original and confirmation sample using an independent samples *t*-test: mean_original_sample_ = 53.625, SD = 4.674, mean_confirmation_sample_ = 50.308, SD = 3.782, *t*_(26.900)_ = 2.397, *p* = 0.024; comparing higher impulsive groups of the original and confirmation sample: mean_original_sample_ = 65.333 SD = 4.703, mean_confirmation_sample_ = 74.792, SD = 5.065, *t*_(38.000)_ = 6.214, *p* < 0.001). See Figure 2 for a plot of the distribution of BIS values in the two samples.

We repeated the identical analyses as described above in the confirmation sample: a moderator analysis with ω as dependent variable, independent variable sum score cognitive capacities, moderator variable impulsivity, as well as age and negative log-likelihood of the hybrid model as nuisance variables was conducted. Confirming the findings in the original sample, we again found a significant interaction effect of impulsivity and cognitive capacities on ω (R^2^ increase due to interaction = 0.081, adjusted R^2^ increase due to interaction = 0.069, *F* = 4.669, *p* = 0.036, Figure 3B). Robust regression based on a bisquare reweighting function confirmed this finding (beta = 0.297, *t* = 2.097, *p* = 0.042). Next, we again tested for the role of the included cognitive subdomains and thus entered the four different test scores (TMT A, TMT B, DS, DSST) as independent variables in separate moderator analyses (dependent variable ω, moderator variable impulsivity). This revealed a significant effect of the interaction “cognitive speed (DSST) by impulsivity” (R^2^ increase due to interaction = 0.111, adjusted R^2^ increase due to interaction = 0.103, *F* = 6.906, *p* = 0.012), and of the interaction “attention (TMT A) by impulsivity” (R^2^ increase due to interaction = 0.080, adjusted R^2^ increase due to interaction = 0.068, *F* = 64.594, *p* = 0.038) on ω. Executive control (TMT B, R^2^ increase due to interaction = 0.048, *F* = 2.685, *p* = 0.109) and working memory (Digit Span, R^2^ increase due to interaction ≤ 0.001, *F* = 0.043, *p* = 0.836) did not significantly interact with impulsivity in their effect on ω. *Post-hoc* regression analyses for both groups (high- vs. low-impulsive individuals) separately revealed that this effect was driven by a significant relation of cognitive speed (DSST) on ω in the high-impulsive group (beta = 0.503, *t* = 2.683, *p* = 0.014); this was absent in the low-impulsive group (beta = −0.126, *t* = 0.629, *p* = 0.536, Figure 4B). *Post-hoc* analyses for TMT A did not indicate a significant effect of TMT A in any of the groups (Beta < 0.309, *t* < 1.649, *p* > 0.110).

## Discussion

In the present study, we did not observe evidence for altered model-free and model-based instrumental control in adult participants with an alcohol-dependent father. Independent of family history, our findings however suggest that an interaction of impulsivity and cognitive capacities influences the degree of model-based decision-making. The latter effect could be confirmed in an independent sample of high and low impulsive individuals (Deserno et al., 2015a).

### Family history of addiction and model-based control

The present work does not provide evidence in favor of a shift from model-based to model-free control in healthy participants with family history of alcohol-dependence. At first glance, this seems in contrast to findings with the same sequential decision task in addicted and other psychiatric patient samples characterized by loss over behavioral control (Sebold et al., 2014; Voon et al., 2015): in these two studies, patients suffering from addictive and other compulsive disorders showed reduced model-based control. It is interesting that, specifically for alcohol-addiction, after a closer look, a more complex picture arises: Voon and colleagues found no reduction of model-based control in alcohol-dependent subjects *per se* but a correlation of model-based control with duration of abstinence (Voon et al., 2015). In the study by Sebold and colleagues, reduced model-based control was found in alcohol-dependent patients overall but effects were attenuated when adjusting for general cognitive functioning (Sebold et al., 2014). Based on the presented null finding in relatives, one might speculate that reduced model-based control arises as a consequence of chronic alcohol-consumption rather than preceding it as a vulnerability factor. Interestingly, Hogarth and colleagues demonstrated that acute alcohol administration indeed leads to reduced goal-directed control in a devaluation paradigm (Hogarth et al., 2012b). Participants showed a reduced effect of devaluation on choice behavior under the acute influence of alcohol compared with placebo.

An additional explanation takes into account our cross-sectional design and the inclusion criteria of unaffected adult participants without any indication of alcohol abuse or other addictive behavior and within an age-range that exceeds the typical onset of addictive disorders. Given this sample selection strategy, participants included in this study might be those who were particularly resilient *not* to develop an addictive disorder—and thus show no alteration in behavioral control. In a previous study, Volkow and colleagues have found putatively protective traits in terms of dopaminergic neurotransmission in a similar sample of unaffected adult relatives of addicted patients (Volkow et al., 2006) and follow the same line of reasoning. To tackle this important question appropriately, longitudinal designs are required to map instrumental control across the developmental process from risk to addiction in adolescence to abstinence and potential relapse in adulthood.

Further, it is to be noted that our study comprised a rather small sample size albeit in a similar range as the previous between-group patient studies (Sebold et al., 2014; Voon et al., 2015). Thus, the null finding of an absent association between family history of addiction requires replication in a larger population, ideally including 1st degree relatives not only of alcohol-dependent subjects, but also of other substance addictions and other psychiatric states characterized by loss of behavioral control like Obsessive-Compulsive Disorder or Binge Eating. In sum, due to the small sample size, the findings of the present study should be interpreted with caution and by no means be taken as finally conclusive. Rather, they are meant to provide a first empirical hint for the field, in order to stimulate further investigation of the theoretically plausible association of addiction vulnerability and the degree of model-based behavior. Recently, online versions of the sequential decision-making task applied have been successfully implemented (Gillan et al., 2015; Otto et al., 2015) which might be a promising venue for testing the research question at hand in larger sample sizes.

### Addiction, cognitive capacities, and instrumental control

Studies suggest that interindividual variability in cognitive capacities relates to a model-based system (Otto et al., 2013a,b; Schad et al., 2014) which was indeed shown to moderate group differences in studies involving patients characterized by cognitive impairment (Sebold et al., 2014). These and our findings suggest that, when observing differences in instrumental control between groups which differ systematically in cognitive factors, one ought to tread carefully when interpreting these; differences might be an epiphenomenon of a more general impairment rather than a specific characteristic for alcohol-dependence. This is also in line with a study using instructed devaluation tasks in alcohol-dependent patients, which revealed a global impairment in learning *per se* (Sjoerds et al., 2013). Here, we replicate the previously reported correlation between cognitive function and model-based control (Schad et al., 2014; Sebold et al., 2014) and find evidence for interaction effects between cognitive function and impulsivity, a recognized risk factor for addiction, on model-based control.

Regarding the correlations between model-based control and general cognition, two aspects should be taken into account. First, as proposed theoretically, model-based computations underlying goal-directed behavior are expensive, thus, they occupy cognitive resources. In this vein, model-free computations underlying habitual behavior can be seen as the cognitively less demanding solution to the task. Indeed, this may bias individuals with relatively higher or lower cognitive capacities *per se* to one or the other way to solve the task. As mentioned before, there is good evidence for such correlations between model-based control in this task and cognitive measures such as cognitive speed and working memory (Otto et al., 2013a, 2015; Schad et al., 2014). Explicitly manipulating cognitive load via a dual task challenge reduces the degree of model-based decision-making in this task (Otto et al., 2013b). In healthy individuals, one can regard this as proof of construct validity that model-based control in this task is indeed a computationally more costly solution. Secondly, all cross-sectional between-group findings with this task in clinical populations related to the degree of model-based control and reduced cognitive capacities are a characteristic of these clinical populations, such as addiction and obsessive-compulsive disorder (Sebold et al., 2014; Voon et al., 2015). Interestingly, reduced model-based control in alcohol addiction compared to healthy controls was indeed related to the reduction in cognitive speed seen in these patients and group differences did no longer reach significance when adjusting for cognitive speed (Sebold et al., 2014). Thus, whether reduced model-based control in patients constitutes a disease-specific mechanism or results from general cognitive impairments can only be teased apart in future longitudinal studies.

### Impulsivity, cognitive capacities, and instrumental control

Interestingly, cognitive dysfunction itself (more specifically, reduced executive functioning), as well as the combination of deficits in cognitive function and impulsivity have been suggested as endophenotypes for drug dependence (Ersche et al., 2012). Thus, our finding of an interaction of cognitive functioning and impulsivity on model-based behavior in two independent samples amends previous studies reporting on an influence of impulsivity on reduced goal-directed control in a devaluation task (Hogarth et al., 2012a) or on accentuated model-free control together with intact model-based control (Deserno et al., 2015a). Our findings in the original investigation and in an independent confirmation sample suggest that the interaction of cognitive capacities and impulsivity plays an important role. Interestingly, in the sample at hand, for which impulsivity measures were not the selection criterion, a positive correlation of cognitive capacity and model-based behavioral control was found in the relatively lower impulsive group. To interpret this finding, one might speculate that relatively, but not extremely low impulsiveness in addition to relatively high cognitive capacities provides an optimal ground for model-based control in this task. In the confirmation sample, which was specifically recruited to consist of a low vs. a highly impulsive group from the extreme ends of the impulsivity measures, impulsivity scores at the higher end matched those of addicted patients. Interestingly, in this sample the correlation of cognitive speed with model-based behavior was driven by the high-impulsive group, suggesting it as a potential compensatory factor in high-impulsive individuals, which were initially assumed to be impaired in goal-directed, model-based behavioral control (Deserno et al., 2015a; Hogarth et al., 2012a).

## Conclusion

In sum, we did not find evidence for an influence of the risk factors positive family history or impulsivity on model-based control *per se*. Due to the limited sample size, this finding has to be interpreted with caution and warrants further investigation in larger sample sizes. Our findings could speak in favor of a *multiple hits* account with different risk conditions playing together to impair or protect model-based behavioral control. Longitudinal designs might help to disentangle these rather complicated interaction effects on model-based control and eventually, on the potential development of addiction.

## Author contributions

AR, LD, and FS designed the study. AR, LD, and TW performed research. AR and LD analyzed the data, AR wrote the initial version of the manuscript. LD, TW, AR, HH, and FS read and corrected versions of the manuscript.

### Conflict of interest statement

The authors declare that the research was conducted in the absence of any commercial or financial relationships that could be construed as a potential conflict of interest.
